# A 24-hour population distribution dataset based on mobile phone data from Helsinki Metropolitan Area, Finland

**DOI:** 10.1038/s41597-021-01113-4

**Published:** 2022-02-04

**Authors:** Claudia Bergroth, Olle Järv, Henrikki Tenkanen, Matti Manninen, Tuuli Toivonen

**Affiliations:** 1 Unit of Urban Research and Statistics, City of Helsinki, Siltasaarenkatu 18–20 A, Helsinki, FI-00530 Finland; 2 Digital Geography Lab, Department of Geosciences and Geography, University of Helsinki, Gustaf Hällströmin katu 2, FI-00014 Helsinki, Finland; 3 Helsinki Institute of Sustainability Science (HELSUS) and Helsinki Institute of Urban and Regional Studies (Urbaria), University of Helsinki, Yliopistonkatu 3, FI-00014 Helsinki, Finland; 4 Department of Built Environment, Aalto University, Otakaari 4, FI-00076 Espoo, Finland; 5 Centre for Advanced Spatial Analysis, University College London, 90 Tottenham Court Road, London, United Kingdom; 6 Elisa Corporation, Helsinki, Finland

**Keywords:** Environmental impact, Interdisciplinary studies, Geography, Society

## Abstract

In this article, we present temporally dynamic population distribution data from the Helsinki Metropolitan Area, Finland, at the level of 250 m by 250 m statistical grid cells. An hourly population distribution dataset is provided for regular workdays (Mon – Thu), Saturdays and Sundays. The data are based on aggregated mobile phone data collected by the biggest mobile network operator in Finland. Mobile phone data are assigned to statistical grid cells using an advanced dasymetric interpolation method based on ancillary data about land cover, buildings and a time use survey. The dataset is validated by comparing population register data from Statistics Finland for night hours and a daytime workplace registry. The resulting 24-hour population data can be used to reveal the temporal dynamics of the city, and examine population variations relevant to spatial accessibility analyses, crisis management, planning and beyond.

## Abstract (summary)

**Table:** 

Measurement(s)	population distribution
Technology Type(s)	mobile phone • digital curation
Factor Type(s)	geographic location • hour of the day • day of the week
Sample Characteristic - Environment	city
Sample Characteristic - Location	Capital Region • Helsinki

Machine-accessible metadata file describing the reported data: 10.6084/m9.figshare.17168978

## Background & Summary

In this paper, we introduce a dynamic population distribution dataset based on mobile phone data from the Helsinki Metropolitan Area in Finland. The mobile phone data are allocated in statistical 250 m × 250 m grid cells using an advanced dasymetric interpolation method^1^ and validated against the population register data from Statistics Finland. Mobile phone data were provided by the largest mobile network operator in Finland. Ancillary data about land cover, buildings and a time use survey were used to estimate the 24-hour population distribution. The resulting dynamic population distribution dataset contains the estimated hourly proportion of population for regular weekdays, and for both weekend days – Saturday and Sunday. Publicly available dynamic population data are provided as a CSV file with unique grid square identifiers for spatial location and hourly population distribution estimates for every hour.

Knowing the whereabouts of people in time and space is necessary to be able to understand how our societies function^2–4^. Accurate information of the actual population distribution and its temporal patterns is of high importance for managing, planning and developing societies from the city to the global level^5^. Knowledge about dynamic population distribution contributes to more effective land use^6^, tourism^7^ and transportation planning^8^, and to more accurate estimation of human pressure on the environment^9^, disease spreading^10^, and exposure of people to and preparedness for disasters^11,12^. Furthermore, it is helpful to optimize public and private sector services^13,14^ and provide insights to various social phenomena, such as socio-spatial inequality and spatial segregation^15,16^.

Currently, information about the actual human presence is often scarce and predominantly based on static population data derived from national population censuses and registers. Thus, these much-used datasets provide knowledge about the “night-time population” rather than the actual presence of people at different times of the day. Also, other static data sources are used to estimate the ambient “daytime population” using dasymetric population mapping approach^17,18^. Nevertheless, census and register data and ambient population modelling neglect the actual setting of temporarily incoming and outgoing population groups, such as tourists and visitors, commuting workers, the short-stay migrant workforce and unregistered people. This could be invaluable information e.g. for mitigating the ongoing COVID-19 pandemic^19^.

In the last two decades, the growing use of novel digital data sources, such as mobile phones, smart cards, social media and other user-generated geographical information has fundamentally changed how population dynamics in space and time can be captured^20^. In particular, mobile phones are considered to be a proxy for people, because they can reveal the precise digital footprints of individuals in space and time^21,22^. In addition, mobile phone data overcomes one of the main weaknesses of traditional data sources for dynamic population mapping – providing a high level of temporal coverage over long time periods^23^. Mobile devices are widely adopted across all population groups and used throughout our daily activities, which allows us to analyse the entire population distributions up to the country level^1,24^, or focus on a certain population group^7,25^. Certainly, it is difficult to obtain datasets from mobile phone operators for use in scientific research and for societal decision making^26^. Our paper describes an open aggregated and dynamic population dataset from the Helsinki Metropolitan Area, based on mobile phone data. We hope that the availability of this dataset facilitates the understanding of our dynamic society^19^ and benefits later analyses for social good, whilst preserving privacy of mobile phone users^19^.

## Methods

### Study area: The Helsinki Metropolitan Area

**Fig. 1:**
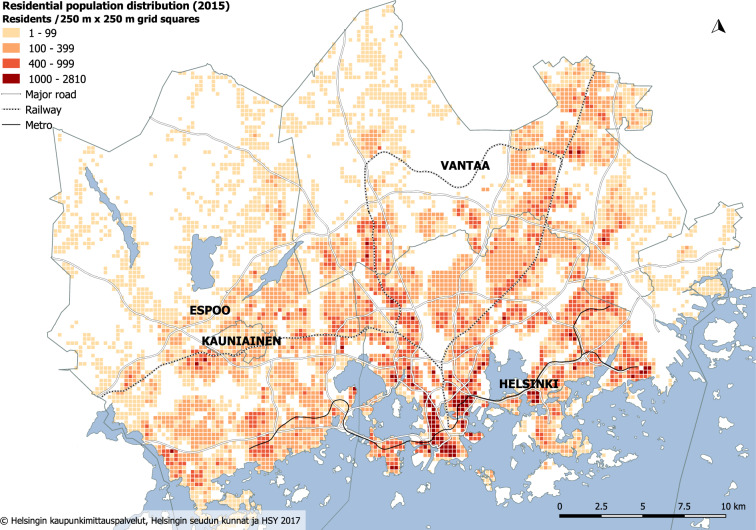
The study area and the number of residents per inhabited 250 m × 250 m statistical grid squares (n = 8,253) in 2015^52^. The data for the background map were obtained from the open Helsinki Region Map dataset^53^.

The dataset covers the Helsinki Metropolitan Area (HMA) in Finland, which consists of four municipalities: Helsinki, Vantaa, Espoo and Kauniainen (Fig. 1). The study area has a population of over 1.1 million inhabitants (1,154,967 on 31.12.2017), which represents roughly one-fifth (21%) of the total Finnish population^27^. The average population density in the study area based on residential data is approximately 1,500 people/km^2^, being the highest in the inner city of Helsinki, which is located on the peninsula in the southern part of the study area.

Mobile phones are used extensively in the study area. At the end of 2017, the mobile phone penetration rate (mobile subscriptions = SIM cards/100 inhabitants) of Finnish households was 126% with approximately 6,960,000 mobile subscriptions^28^, which is above the global and the European average rates – 103.6% and 120.4%, accordingly^29^. It is estimated that 89% of 16–89-year-olds own a smartphone in the Finnish capital region^30^. The results of the survey suggest that there is no significant difference between women and men in terms of the phone ownership or use. A survey done in 2017 from the study area shows that 69% of 7-year-old children already have their personal mobile phone^31^. At the end of 2018, Elisa Oyj has the largest market share of mobile subscriptions (38%) in Finland followed by Telia Finland Oyj (33%) and DNA Oyj (28%)^32^.

### Data processing steps – flowchart

**Fig. 2:**
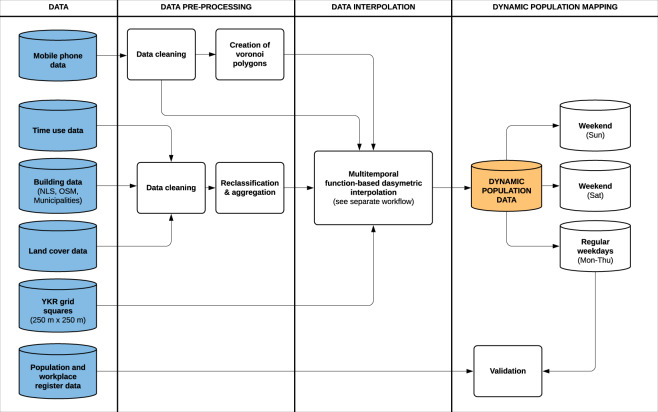
The general workflow of the study. The steps of the multi-temporal function-based dasymetric (MFD) interpolation method are presented in more detail in Fig. 5.

Producing the data required various processing steps. First, we pre-processed the raw data by cleaning, reclassifying and aggregating the data into polygons representing the approximated coverage areas of the operator base stations. Secondly, we used the pre-processed data as input to estimate hourly weekday population distribution in the study area by applying a dedicated dasymetric interpolation method to enhance the spatial accuracy of the mobile phone data. We calculated the hourly weekday (Monday-Thursday), Saturday and Sunday population distribution using a network-driven mobile phone dataset defined as High-Speed Packet Access (HSPA) calls (see details below). Friday was left out since time use patterns of people on Friday typically deviate from the other non-weekend days. We validated the data against the official population register data representing the residential population and workplace data. Finally, we packaged and visualized the data to provide an understanding of the dynamic population. The steps in the empirical study were conducted primarily using Python for analysis and QGIS for visualizing the results. The workflow of the study is illustrated in Fig. 2.

### Mobile phone data

Network-driven mobile phone data from a two-and-a-half-month study period from late October 2017 till early January 2018 provided by the Elisa Oyj mobile network operator (MNO) was used to map the dynamic population distribution in the study area. More specifically, we use HSPA (High-Speed Packet Access) call data which are automatically collected and pre-calculated key performance indicator (KPI) for data transmission by users in the mobile network based on the standard principles introduced by 3 GPP^33^. Since HSPA data are calculated based on radio network counters there are no identifiers or links to any mobile device nor personal information. As the HSPA data are inherently anonymous, there is no opt-in or opt-out possibility. Thus, all the mobile devices connected to the network are in scope, including foreign mobile devices using roaming services.

The mobile phone data used was passively (automatically) collected and processed by the MNO prior to providing us with the data. First, the MNO aggregated the set of raw counters used to calculate HSPA calls from antenna (cell) level to base station (site) level before calculating the actual KPI for each base station according to the principles defined by 3 GPP^33^. The raw counters as well as the data at base station (BS) level had the temporal accuracy of one hour. Second, the BS coordinates of the base stations equipped with multiple directional antennae, were approximated using the coordinates of the antenna with the maximum X and Y coordinate value by the MNO. This only has an impact on the spatial accuracy of the BS coordinates when the antennae were not attached to a mast-like cell tower.

Finally, for reasons of business confidentiality, a randomized error of up to ±100 metres was set to BS coordinates in the inner city of Helsinki by the MNO before providing us the data. That is, each coordinate pair is randomly relocated within the range between −100 metres and +100 metres from the original location. Outside the inner city, the error was set up to ±200 metres, accordingly. In general, the spatial accuracy of the data is dependent on the density of the base station network (highest in the city centre and other densely populated areas, where use rates are highest)^1,34^. The median theoretical coverage area based on Voronoi polygon modelling in our study area is 0.24 km^2^.

### Content of the raw data

The original dataset contained approximately 3.8 million rows of data and covered all base stations by the given operator in the Uusimaa region in Southern Finland. The original dataset received from the MNO contained six attributes: the hourly count of HSPA calls, the identifier of a base station and data record, geographical (X, Y) coordinates (in ETRS-TM35FIN coordinate system) and timestamp with an hourly precision (YYYY-MM-DD hh) (Table 1).

**Table 1:** Illustration of the original dataset received from the MNO including six variables.

ID	Timestamp	Base Station ID	HSPA Calls	X	Y
1	2018-11-15 08:00:00	000001	145	369876	6671234
2	2018-11-15 08:00:00	000002	982	368765	6672345
3	2018-11-15 08:00:00	000003	545	366543	6673456
4	2018-11-15 09:00:00	000001	320	369876	6671234

To contextualize the HSPA call data, it is a collection of downlink (HSDPA) and uplink (HSUPA) protocols, which enables faster data transmission in a Universal Mobile Telecommunications System (UMTS) cellular network^35^. In general, radio access bearers (RAB) are responsible for transmitting voice or data in 3G telecommunication networks, but if HSPA is supported by the network, data transfer can be replaced by HSPA bearers when prompted by HSPA call requests^35^. Thus, the HSPA calls in the dataset encompass the majority of 3G mobile data transmissions. Data transfer from 4 G networks was, however, not available for the study.

### Temporal distribution of the raw data

**Fig. 3:**
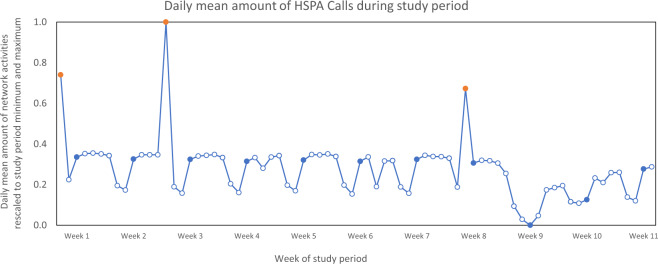
Daily mean number of HSPA calls during the study period. The values have been rescaled using min-max normalization. Blue markers indicate Mondays. Days with abnormal data are highlighted (indicated with orange markers) and excluded in producing the dynamic population dataset.

The HSPA call data show clear temporal patterns both at weekly and daily levels. Regarding the whole study period, a recurring weekly rhythm can be distinguished (Fig. 3). The amount of network activity is relatively similar between the weekdays from Monday to Friday, which decreases during the weekend, with the lowest rates on Sundays. The weekly pattern is disrupted during the holiday season with lower mobile phone usage compared to the day of the week average. Examples include Finland’s Independence Day (6.12.), New Year’s Day and Christmas Day. Days with abnormally high values are system biases inherent in the raw dataset.

**Fig. 4:**
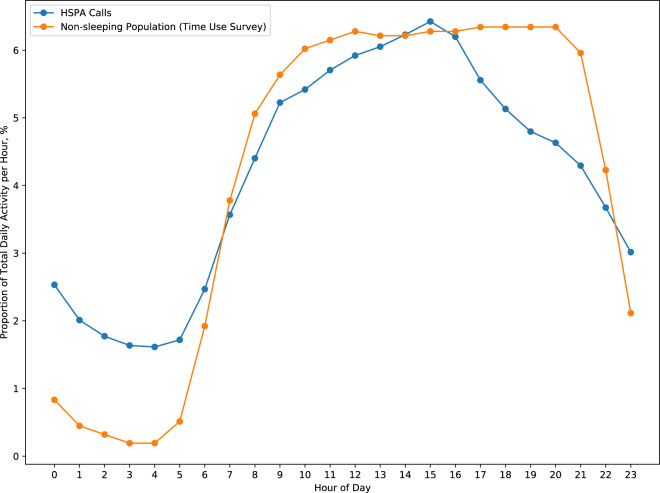
Daily temporal patterns of mobile phone data use and statistical non-sleeping population on an average weekday (Monday–Thursday) in HMA.

There is a distinct pattern in the temporal distribution of network activities, even at the diurnal level. On a regular workday (Monday–Thursday), mobile phone data follow a similar pattern as shown in the activities of people from the Time Use Survey with lowest values during the night, from 00:00 to 05:00 and more evenly distributed over the course of the day (Fig. 4).

### Pre-processing of mobile phone data

**Fig. 5:**
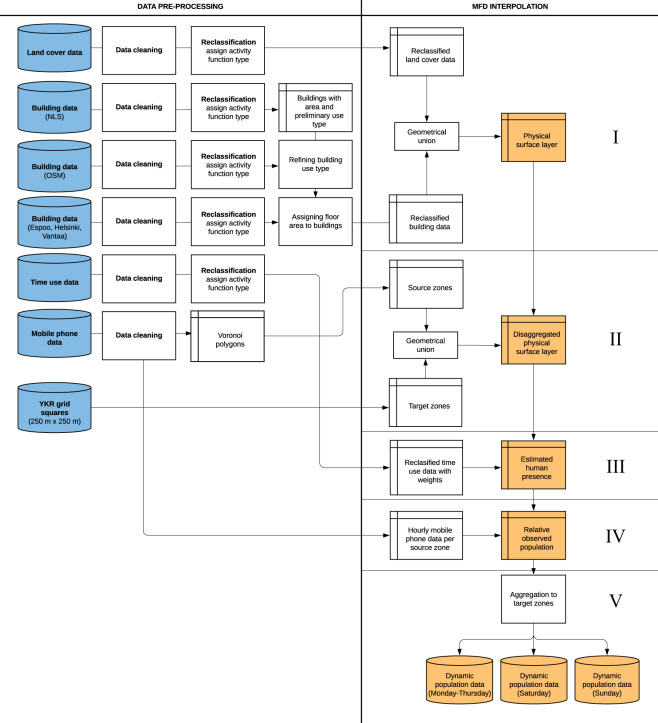
General workflow of the multi-temporal function-based dasymetric (MFD) interpolation method. Steps I–V indicate the phases of the MFD interpolation method. Original input data sources are shown in blue and study outputs in yellow.

The mobile phone data were prepared for constructing the dynamic population by filtering, cleaning, manipulating and aggregating the original data (see Fig. 5). We excluded days (n = 3) with abnormal data (Fig. 3) and any hourly values (incorrect or missing data from a base station) that might distort the results. We further cropped the data to the extent of the study area, removed a handful of base stations with no activity during the whole study period (or if two base stations had identical ID in different locations), and merged a few base stations with identical coordinates. We also filtered out duplicate hour entries caused by the transition to winter time.

After cleaning and editing the data, we filtered the data to present regular workdays (Monday-Thursday), and separately both weekend days – Saturday and Sunday due to distinctive temporal activity pattern. After the filtering, 62 days were left for further analysis, out of which 42 between Monday and Thursday and 10 on both Saturdays and Sundays. Finally, we aggregated the data to get the median number of HSPA calls during the study period for every base station (BS) for every hour of the day.

### Constructing the dynamic population from mobile phone data

**Fig. 6:**
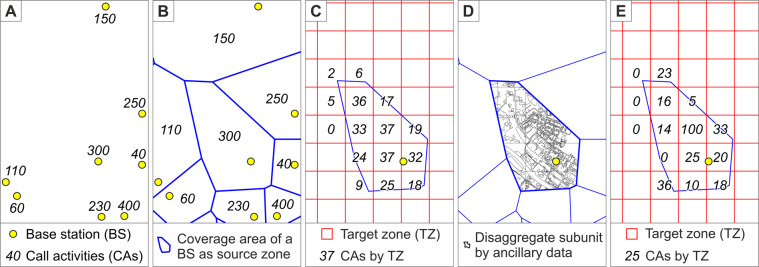
Workflow of the MFD interpolation (adopted from Järv *et al*.^1^). The (**a**) mobile phone data as points representing the base stations, (**b**) Voronoi polygons as theoretical coverage areas of the base stations (**c**) aggregation to the target zones based on their relative size, (**d**) integration of ancillary data and (**e**) final interpolated data.

To distribute the mobile phone data from the base stations to the statistical grid squares, we used the *multi-temporal function-based dasymetric (MFD) interpolation* method^1^, see Figs. 5 and 6. The MFD method is a *dasymetric interpolation* method belonging to the same family of *areal interpolation* methods as areal weighting. However, dasymetric interpolation differs from areal weighting because it uses ancillary data to improve the interpolation of data from existing spatial units (i.e. *source zones*) to desired spatial units (i.e. *target zones*). This approach has been regarded as one of the most feasible methods for refining the spatial resolution of population and has been widely applied in different application fields^17,18^.

The datasets used for preparing the dynamic population distribution using a dasymetric interpolation method are listed in Table 2.

**Table 2:** Data sources used for the dasymetric interpolation of mobile phone data. The source for each dataset is provided as a hyperlink.

Dataset name	Description/source	Source (Year)	Phase of analysis	Open data
Mobile phone data	Hourly network-driven mobile phone data records aggregated on BS level (contact: matti.manninen@elisa.fi).	Elisa Oyj (2017–2018)	Extracting spatio-temporal population patterns	No
Time use data	The average time use by activity and location on 10 min intervals in HMA. The data is based on a decennial time use survey.	Statistics Finland (2010)	Creating a probability matrix for human presence	No (for a fee)
Land cover data	Corine Land Cover 2012 raster (20 m × 20 m). Updated every six years.	Finnish Environment Institute (2012)	Allocating mobile phone data to land use areas based on time use data	Yes
Building data	Building polygon footprints and use type from the national topographic database. Updated annually.	National Land Survey of Finland (2018)	Allocating mobile phone data to buildings based on time use data	Yes
	Building polygon footprints, floor area and floor count of buildings in Helsinki. Updated daily.	City of Helsinki (2018)	Assigning floor area to buildings	Yes
	Building polygon footprints, floor area and floor count of buildings in Espoo. Updated weekly.	City of Espoo (2018)	Assigning floor area to buildings	Yes
	Building polygon footprints, floor area and floor count of buildings in Vantaa. Updated weekly.	City of Vantaa (2018)	Assigning floor area to buildings	Yes
	Buildings polygon footprints and use type of buildings.	OpenStreetMap (2018)	Refining functionality type of buildings	Yes
YKR grid squares	Polygon layer of 250 m × 250 m grid squares (n = 13,231)	Digital Geography Lab, Statistics Finland (2015)	Target zones for refining mobile phone data	Yes
Grid Database 2016	Registry-based residential population data (2015) and workplace data (2014) on 250 m × 250 m statistical grid cells	Statistics Finland (2014–2015)	Evaluation of dynamic population data	No (for a fee)

### Creation of the physical surface layer

In the first stage of the MFD method, land cover and building data were pre-processed and combined to create *the physical surface layer* which is a spatial layer representing land use information including a vertical dimension (building volumes). It is used as an input data for calculating the likelihood of human presence at the later stages of the MFD^1,36^. Each feature in the physical surface layer was assigned an activity function type, which enabled us to further link the data with the time use survey data (Table 3).

**Table 3:** The division of activity function types linked to reclassified land cover and building data, and to time use survey data.

Activity Function Type (AFT) a	Time Use Data	Land Cover Data	Building Data
Residential	at home or accommodation	residential area	residential and leisure building, hotel, prison
Work	at work or school	industrial and commercial area, construction site	office, industrial building, school, public building not for in situ services (e.g. hospital, city hall)
Retail & Service	shopping or using services	—	shopping centre, services (e.g. hairdresser, gas station)
Transport	travelling	transport network (e.g. road, harbour, parking space)	Passenger terminal, station building
Restricted	(no activity)	water, wetland, arable land, dump site, restricted area	—
Other	other activity, e.g. leisure activity	other areas, e.g. forest, park, cemetery, sport and leisure facility	other building e.g. religious building, restaurant, entertainment, public building for in situ services (e.g. library, sport facility)

The classification is adopted from Järv *et al*.^1^.

^a^Target class, common key.

Regarding the land cover data, we used a country-specific CORINE Land Cover raster dataset (the most recent version of it at the time) with a spatial accuracy of 20 m × 20 m to determine the land cover classes of the study area^37^. The spatial accuracy of the more broadly available Pan-European CORINE Land Cover vector dataset was too coarse (25 ha) for the study purposes. Similarly, the more recent openly available land cover data provided by the National Land Survey of Finland and the Helsinki Region Environmental Services Authority HSY were not applicable due to too low spatial accuracy. The refined land cover classification enabled us to link land use classes to activity types in the time use data.

**Fig. 7:**
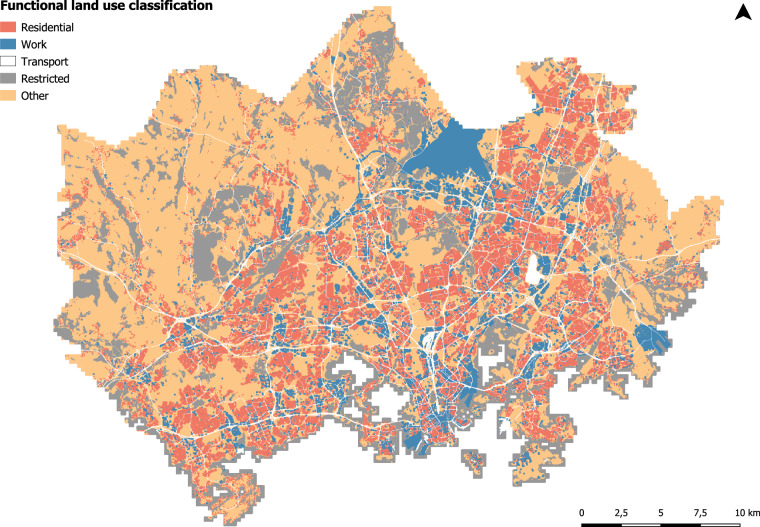
The reclassified land cover dataset based on CORINE Land Cover 2012.

To prepare the land cover data for the MFD method, the dataset was transformed into vector format, reclassified and cropped to the extent of the study area. Like Järv *et al*.^1^, the land cover data were reclassified from the original classes (n = 48) to five classes based on their activity function types: (1) residential, (2) work, (3) transport, (4) restricted and (5) other (see Bergroth p.58^38^; Fig. 7). To improve the classification, both the international Helsinki-Vantaa airport area (mid-north; Fig. 7) and the Vuosaari cargo harbour area (east) were reclassified from transport class to the work class as an important site for workforce due to their work-driven functions.

**Fig. 8:**
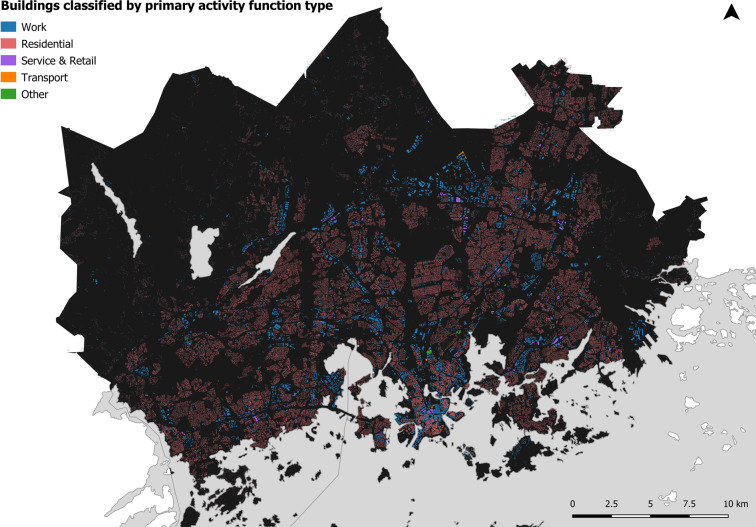
The reclassified buildings dataset (n = 153,357) based on their primary activity function type.

In terms of the building data, building polygons were extracted from the National Topographic Database^39^. In total, 160,490 buildings were located in the study area. The building data were cleaned by calculating the area of each building footprint and filtering out buildings with an area below 20 m^2^ (n = 6,860) leaving 153,357 buildings left for further analysis. Similarly with Järv *et al*.^1^, the buildings were first classified into three types according to their primary activity function type – residential, work and other buildings (see Bergroth p. 58^38^). Here, non-classified buildings were assumed to have work as the main activity function (i.e. work buildings), given that the dataset has accurate classification for buildings that have primary activity functions associated with residential and other activity, but not for work activity function. To further enrich the data and refine the classification, we retrieved additional building information from OpenStreetMap (n = 72,574)^40^. Using the OpenStreetMap data, the building classification was expanded to cover also retail and service and transport activity function types, which could not be extracted from the original building data (see Bergroth p. 140^38^; Fig. 8).

Only one activity function type was assigned to each building. We recognize the crudeness of the selected approach as buildings may have multiple use types either simultaneously or at different times. However, the current level of accuracy is expected to be feasible for the purpose of this study. The final classification of buildings per activity function type is presented in Fig. 8 and Table 4.

**Table 4:** Reclassified land cover and building data used in the MFD interpolation per activity function type.

Activity Function Type	Land cover Area (km^2^)	Buildings (count)
Residential	161.79	104,988
Work	81.35	47,268
Service and Retail	0	302
Transport	43.54	55
Restricted	144.54	0
Other	406.02	744
Total	837.24	153,357

The physical surface layer also takes into account the vertical dimension in the likelihood of human presence. To retrieve the vertical dimension, we used information about building footprints, floor area (m^2^) and floor counts based on national building registers (not available for the city of Kauniainen). The municipal data were further cleaned, combined and joined to the original building dataset. Finally, a geometric union was performed to combine the reclassified building and land cover layers.

### Spatial disaggregation by the source and target zones

After creating the physical surface layer, a geometric union was performed between the physical surface layer, source zone and target zone layers to create *the disaggregated physical surface layer* – a layer where physical surface layer units are divided into subunits so that each subunit (referred as *s* in Formulas, below) is designated both to one unique source zone (*j*) and one unique target zone (*z*), see Fig. 6. In general, any spatial division can be used regarding the source zones and target zones. Voronoi polygons were used to estimate the theoretical coverage areas of base stations (source zones), and 250 m × 250 m statistical grid cells were used as the target zones^41^. As a result, the study area was divided into 345,917 subunits, each with a designated activity function type and spatial unit type (building or land) as well as floor area. The area of each subunit was recalculated after the overlay operation.

Next, the *relative floor area* of each subunit was calculated to include the vertical dimension in the interpolation. First, the absolute floor area was assigned to the subunits based on their spatial unit type and activity function type. For subunits with the spatial unit type ‘land’, the geometric area of the subunit was set as the floor area. For subunits with the spatial unit type ‘building’, the floor area was based on openly available building data from the municipalities of Espoo^42^, Helsinki^43^ and Vantaa^44^ containing the building register-based floor areas and floor counts. The use of actual floor areas provides a more accurate estimate than the LiDAR-based approach applied in Järv *et al*.^1^, in which the floor area was estimated from the building height extracted from the digital surface model (DSM).

In case the building register data were not openly available (e.g. in Kauniainen), the floor area was estimated based on the actual or mean floor count and a specific floor area coefficient. The mean floor count was 2 for residential, service and retail buildings, and 1 for others. The floor area coefficient was 0.95 for residential buildings, 0.91 for service and retail buildings, and 0.98 for other buildings. The floor area coefficient was calculated as the median ratio between the actual floor area and the product of the building footprint area and the floor count. Both the mean floor count and the floor area coefficient were calculated separately for buildings of each activity function type. Finally, the relative floor area (RFA) was calculated for each subunit within a source zone, based on the Formula 1:

1 $$RF{A}_{s}^{j}=\frac{F{A}_{s}^{j}}{\sum F{A}_{s}^{j}\in j}\forall s\in j$$

where

*RFA* = relative floor area

*FA* = floor area

*s* = spatial subunit

*j* = source zone

As a result, the sum of the relative floor area of all subunits within one source zone (Voronoi polygon) equals to 1. The higher the relative floor area of the subunit, the higher the likelihood that activity is allocated to that subunit.

### Integration of the temporal human activity data

In the third phase of the MFD method, time use data were used to integrate the physical surface layer to create a probability matrix for allocating the mobile phone data to target zones within each source zone. As a result, each spatial subunit got an hourly likelihood rate of human presence based on its activity function type.

**Fig. 9:**
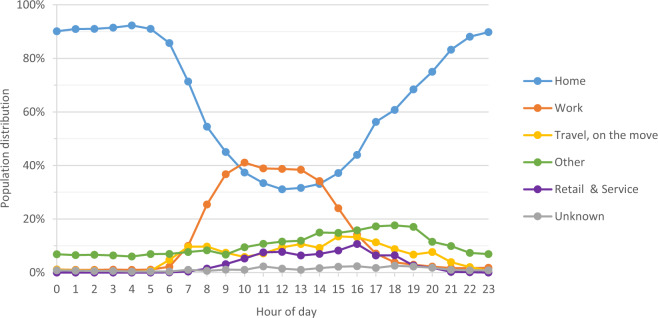
Time use by location and activity per hour on weekdays (Monday-Thursday) of over 10-year-olds in the Finnish Capital Region 2009–2010^45^.

The *estimated human presence* (EHP) in each subunit was calculated using human activity data based on the latest Finnish time use survey^45^ carried out in 2009, according to the guidelines for Harmonised European Time Use Surveys (HETUS) issued by Eurostat. The time use survey allows for the calculation of the human activity data for each hour based on the activity location of over 10-year-olds in the HMA (Fig. 9).

To calculate the estimated human presence, we first aggregated the human activity to the hourly level. Second, we reclassified human activity from the survey to the following classes based on the location, where the activity was undertaken to join it with the physical surface layer: 1) residential, 2) work (incl. education), 3) transport, 4) retail and service, 5) unknown and 6) other (such as recreational areas) (see Bergroth p. 139^38^).

An *hourly probability coefficient* (H) was assigned to every hour of the day based on the time use data. In addition, a *seasonal probability coefficient* (M) was assigned to account for the impact of the season on the distribution of people indoors and outdoors. According to a study conducted by Hussein *et al*.^46^, people were found to spend approximately 90% of the day indoors in Helsinki during the winter and spring. Similarly, as in Järv *et al*.^1^, the results are assumed to be suitable for the dasymetric interpolation, since the mobile phone data used for estimating the population distribution were also collected during winter. The seasonal factor was applied for three of the activity function types (residential, work and education, other). Thus, a subunit of the work activity function type would receive a coefficient of 0.9 if the spatial unit type was ‘building’ and a coefficient of 0.1. if the spatial unit type was ‘land’. Subunits with the other activity function types were assigned a factor of 1, except restricted areas, which were assigned a factor of 0. This way, the MFD method prevents population being allocated to a subunit of a restricted type. Overall, the estimated human presence per every spatial subunit at a given time unit (hour) was calculated using Formula 2:

2 $$EH{P}_{s}^{j,t}=\left[{H}_{a,u}^{t}\times {M}_{a,u}\right]\times RF{A}_{s}^{j}$$

where

*EHP* = estimated human presence

*t* = time unit

*H* = hourly factor

*M* = seasonal factor

*RFA* = relative floor area

*a* = activity function type

*u* = spatial unit type (building or land)

*s* = spatial subunit

*j* = source zone

### Integration of the mobile phone data

In the fourth phase of the dasymetric interpolation, the mobile phone data, were integrated to the physical surface layer enriched with hourly and seasonal human activity data. The mobile phone data containing the hourly median number of the different network activities were linked to the physical surface layer based on the BS identifier. First, the mobile phone activity per spatial subunit was normalized by dividing it by the sum of the corresponding value of all spatial subunits in the study area. Hence, the sum of the relative proportion of mobile phone data of all subunits in the study area is 1. The *relative proportion of mobile phone data* per spatial subunit of study area total at given hour was calculated using Formula 3:

3 $$RM{P}_{s}^{j,t}=\frac{M{P}_{s}^{j,t}}{\sum M{P}_{s}^{j,t}\in S}$$

where

*RMP* = relative proportion of mobile phone data

*MP* = mobile phone data

*s* = spatial subunit

*t* = time unit

*j* = source zone

*S* = study area

The formula was calculated separately for each of the three weekdays – regular workday (Monday – Thursday), Saturday and Sunday. Secondly, the hourly normalized mobile phone data for each weekday were multiplied by the hourly estimated human presence to allocate the population to the subunits based on the physical surface layer and time use statistics. The *relative observed population* was calculated using Formula 4:

4 $$RO{P}_{s}^{j,t}=EH{P}_{s}^{j,t}\times RM{P}_{s}^{j,t}$$

where

*ROP* = relative observed population

*EHP* = estimated human presence

*RMP* = relative proportion of mobile phone data

*s* = spatial subunit

t = time unit

j = source zone

### Spatial aggregation to target zones

In the fifth and final phase of the MFD method, the spatial subunits were aggregated to the statistical 250 m × 250 m grid cells (n = 13,231). The aggregation was performed by dissolving the subunits based on the target zone ID. As a result, each target zone was assigned the sum of the relative observed population of all spatial subunits within the given target zone. The aggregation to the target zones can be summarized as follows (Formula 5):

5 $$ZRO{P}^{z,t}=\sum _{s\in z}RO{P}_{s}^{z,t}$$

where

*ZROP* = spatially aggregated relative observed population per target zone

*ROP* = relative observed population

*t* = time unit

*s* = spatial subunit

*z* = target zone

As a final result of the MFD method, three normalized population data layers for each hour of the day for regular workday (Monday – Thursday), Saturday and Sunday were created. After normalization, the sum of all values for each one-hour period equals to 100 (i.e. 100% of total population). The script used to run the MFD method is based on Järv *et al*.^47^ and openly shared via GitHub: https://github.com/DigitalGeographyLab/mfd-helsinki.

## Data Records

The dataset comprises of three files named “HMA_Dynamic_Population_WorkingDay” for representing dynamic population distribution during working days from Monday to Thursday, “HMA_Dynamic_Population_Saturday” for representing dynamic population distribution during Saturdays, and “HMA_Dynamic_Population_Sunday” for representing dynamic population distribution during Sundays. All three files are stored in a CSV file format. The dataset is openly available from Zenodo licensed under Creative Commons Attribution 4.0 International^48^. The three datasets include the same data structure as presented in Table 5.

**Table 5:** The attributes of the dataset.

Field name	Description
YKR_ID	Unique identifier for each statistical grid cell (n = 13,231). The identifier is compatible with the statistical YKR grid cell data by Statistics Finland and Finnish Environment Institute.
H0, H1, H2 … H23	The proportion of population within a grid cell from the total population in the study area during a one-hour period. In total, 24 fields formatted as “Hx”, where x stands for the hour of the day (values ranging from 0–23). For example, H0 stands for the first hour of the day: 00:00–00:59. The sum of all values for each one-hour period equals to 100 (i.e. 100% of total population).

## Technical Validation

### Validation methods

One of the common ways to validate the feasibility of the population distribution derived from the mobile phone data as a proxy for people, is to compare them against the night-time population provided by residential population data^1,24,49^. Previous studies have slight differences in defining the night period, thus, we selected the night-time window (02:00–04:59) based on the hours when people are most likely to be at home according to the time use survey^45^. We validate the night population (02:00–04:59) against the official population register data, and the day population (15:00–15:59) against the official workplace register data as the best available proxy for estimating the daily population distribution. We measure the number of residents (the night-time) and workplaces (the daytime) at 250 m × 250 m statistical grid cell level.

The population distribution derived from the mobile phone data was validated using four evaluation indicators that have been used before in evaluating dasymetric population mapping^1,50^: (1) Pearson correlation coefficient and (2) standard error (SE), (3) mean absolute error (MAE) and (4) coefficient of variation (CV). The correlation was calculated for each hour of the regular working day and separately for the night-time period. The latter two indicators are measured only for the night-time period. The coefficient of variation (CV) is calculated by dividing the root mean square error (RMSE) by the overall number of population in the study area^1,50^.

Finally, we analysed the feasibility of the population distribution derived from the mobile phone data by evaluating the distribution of population by the activity function type class (residential, work, other, transport, service and retail, restricted) at spatial subunit level (see, Formula 3). We compared the distribution against Time Use Survey data (Fig. 9) for both the night and the day populations.

### Validation results

**Fig. 10:**
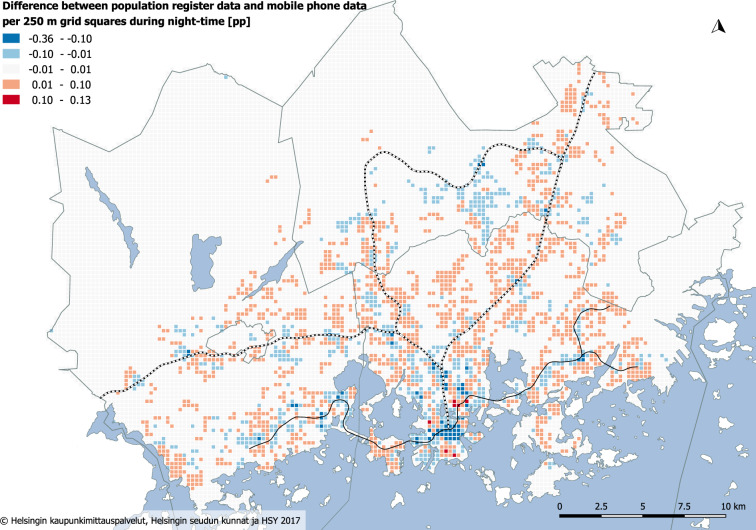
Absolute differences in regular workday population distribution (percentage points, pp) between population register and mobile phone data during night-time (02:00–04:59). Blue colour indicates grid cells, where the population register underestimates the actual population compared to the mobile phone data.

The geographical comparison between the spatial distribution of the population based on interpolated mobile phone data and the population register data uncovers distinct differences between the datasets at night (02:00–04:59). The population register data tend to underestimate the actual population revealed by mobile phone data, especially in non-residential areas – the Helsinki city centre, the Helsinki-Vantaa international airport district, and the Pasila-Ilmala logistic and office district (Fig. 10). These areas have night-time work and service functions, in addition to late-night entertainment at the city centre. In contrast, the population register data overestimates the proportion of present population mostly in residential areas as not 100% of population is always present at home at night.

The night-time population distribution derived from interpolated mobile phone data is strongly correlated (ρ = 0.683) with official population register data (Table 6), which is almost identical with the finding of Järv *et al*.^1^. Also, the SE of the linear regression, MAE and RMSE coincide with the findings of Järv *et al*.^1^.

**Table 6:** Statistical evaluation of the night-time population distribution based on interpolated mobile phone data against official population register data.

Method of Evaluation		
Linear Regression	Correlation Coefficient (Pearson)	0.683***
	Standard Error (SE)	0.007178
Mean Absolute Error (MAE)		0.000052
Coefficient of Variation (based on RMSE)		0.000147

***Correlation is significant at the 0.001 level (2-tailed).

**Fig. 11:**
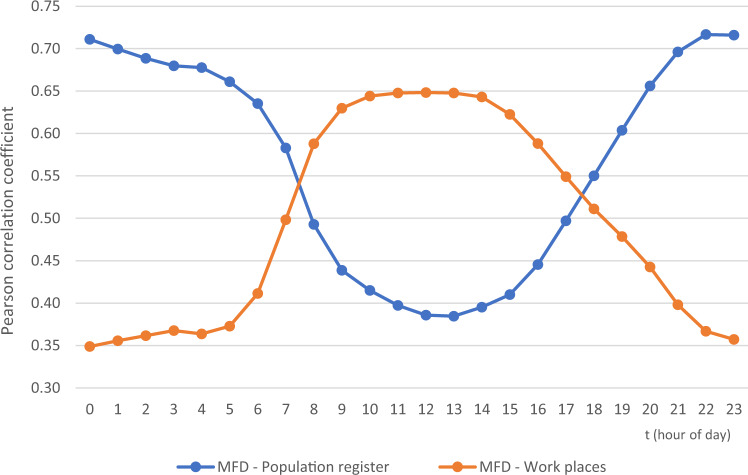
Hourly correlation coefficients of a regular workday (Monday – Thursday) between population distribution from interpolated mobile phone data and official register data – population register (blue) and workplace register (orange). All correlations are significant at the 0.001 level (2-tailed).

Figure 11 shows the correlation between the interpolated mobile phone data and residential population for each hour of the regular workday. The hourly correlation varies significantly during the day. The correlation coefficient of individual hours is strongest between 22:00 and 00:59 (ρ > 0.7) and weakest during midday from 11:00 to 13:59 (ρ < 0.4). In contrast, the correlation between the interpolated mobile phone data and workplaces (proxy for daytime population) is the weakest during the night from 21:00 to 05:59 (ρ < 0.4) and strongest during midday from 09:00 to 14:59 (ρ > 0.6).

**Fig. 12:**
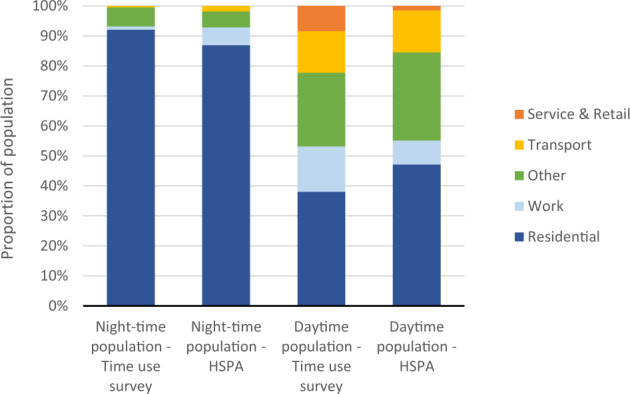
Population distribution of the reallocated population by activity function type during night-time and daytime compared to the original time use survey data.

Finally, the distribution of population by the activity function type between the interpolated mobile phone data and time use survey data shows clear similarities (Fig. 12). During the night-time, the MFD interpolation allocates a significant proportion of the population based on mobile phone data to residential areas and only 13% of the population to elsewhere (areas with the function type of work, transport and other). According to the time use survey, some 92% of the population are located in residential areas. During the daytime (15:00–15:59), the population based on mobile phone data is allocated more to areas with residential and work functions, but to a lesser extent to areas with service and retail functions, and other functions, compared to the time use survey data. One needs to acknowledge that activity function types from the interpolation model consider only one activity function per spatial subunit and not mixed functions, which may include some minor biases compared to the time use survey.

Overall, the evaluations against the best possible comparison data above shows the applicability of the interpolated mobile phone data as a proxy for temporally dynamic population distribution in the Helsinki metropolitan area in Finland. Certainly, one challenge in validating the *de facto* dynamic population is the absence of ground truth data against to which mobile phone data can be validated^1^. The official population register data provides an excellent baseline to evaluate a night-time population. However, even during the night-time, not all people are always at home (see time use survey data in Fig. 12). Yet, the biggest challenge is to evaluate the dynamic population during the daytime – there are no reliable data to compare with. The best available register data is official workplace register data as a proxy for indicating the dynamic population. Certainly, it is possible to combine workplace information with school and university enrolment information, but we would still miss people conducting other activities and those temporarily on the move (e.g. tourists) that would raise the validity question about the ground truth data, per se.

One inherent weakness of the HSPA call data used in this study, is the fact that it only includes data via 3 G technology, whereas increasingly more data transfers in the mobile networks are done via 4 G and 5 G technology. Yet, we assume that the possible bias should not be significant from a spatial and temporal perspective. Hence, we believe this bias occurs equally throughout the space and time and does not affect the interpolation of dynamic population distribution.

## Usage Notes

The presented mobility dataset can be used for a range of applications as it can be directly linked to the official statistical grid data, as well as to other datasets produced with the same grid system, such as a data release^51^ representing travel time and distance information by different travel modes (private car, transit, bike, walking). Combined, these datasets allow e.g. dynamic accessibility modelling for the region, similarly to Järv *et al*.^13^.

Below, we introduce a few examples demonstrating how the dynamic population distribution data can be used to understand population dynamics. We further exemplify how it can be linked to travel time data in HMA to study grocery shop accessibility considering dynamism in mobility, accessibility and activity locations.

### Inspecting the dynamic population on a local level

**Fig. 13:**
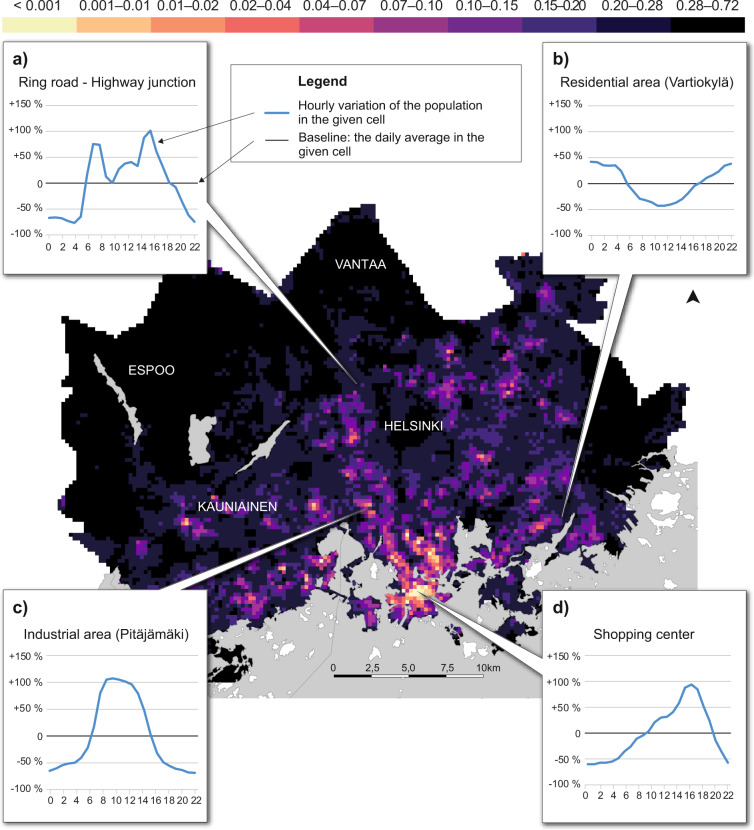
Map of hourly distribution of the population between 12:00–13:00 in HMA during a regular workday. Graphs a, b, c and d represent the variation of the population in a given statistical grid cell at different hours of the day from the daily average.

Various spatiotemporal patterns of population distribution can be extracted from the mobile phone data in the HMA on an average weekday. The map in Fig. 13 shows how the population is distributed in the study region between 12:00–13:00 during weekdays. The graphs illustrate how the active population in the grid square fluctuates during the hours of the day in four locations, showing how areas with distinct functions have distinct temporal population patterns. The locations represent four functionally different areas in the urban structure: (a) transport, (b) residential use, (c) work and (d) service and retail. The population in a typical transportation area (a) shows clear peaks during the rush hour times in the morning, at midday and in the late afternoon, whereas residential areas (b) have a u-shape pattern with the highest proportion of population present at night and in the evening, and lowest at midday. The relative population concentration in a working/industrial area shows the highest activity rates between 08:00 and 16:00 following standard working hours, while the temporal pattern in the shopping centre area has the highest peak in the evening when people go for shopping after the work. Hence, the data can reveal (or confirm) various interesting aspects about the dynamics of societal functions in the study region.

### Inspecting dynamic accessibility of grocery shops

When combined with dynamic travel time data^51^, it is possible to estimate e.g. how many people can reach a given grocery shop at different hours of the day considering temporal changes both in the whereabouts of the population and the travel times by different modes of travel. Following the conceptual framework of dynamic accessibility presented by Järv *et al*.^13^, we investigated how the temporal variation in i) people’s locations, ii) travel times by public transport, and iii) opening hours, influence the location-based accessibility of grocery shops (activity locations) in the Helsinki Metropolitan area. The locations and opening hours of the grocery shops were collected from the websites of the shops. To consider the dynamism of the transportation, we use transit route and schedule data provided by local transportation authorities, as well as walking paths extracted from OpenStreetMap (see^51^ for methodological details). Finally, the temporal variation in the locations of people were derived from the 24-hour population distribution dataset extracted from the mobile phone data introduced in this study. We selected two grocery shops as a case study, which represent the perspective of a local small grocery shop. Shop 1 is located in a neighbourhood next to a major workplace area in the inner city of Helsinki, whereas shop 2 is located on the fringe of the study area in a residential-driven neighbourhood (in the city of Vantaa). Grocery shops were selected as an example, because it is important to access them regardless of the time of the day, for example from the perspective of night workers.

**Fig. 14:**
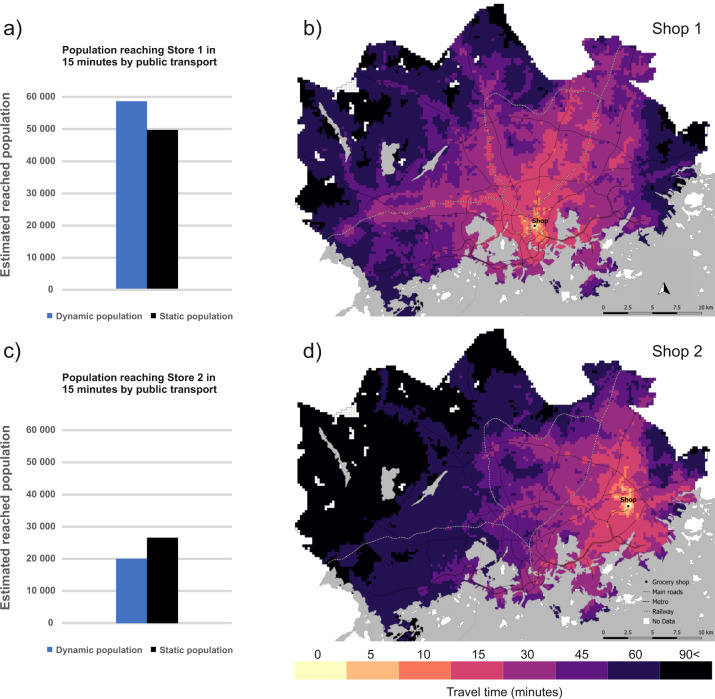
Population accessing grocery Shop 1 (above) and Shop 2 (below) in 15 minutes by public transport between 17:00–18:00. The number of reached population is proportional to the number of inhabitants in the HMA (1,154,967 on 31.12.2017). In the case of Shop 1, the static population data underestimates the number of people reaching the shop by approximately 9,000 individuals (**a**), whereas in the case of Shop 2, the static population overestimates the reached population by approximately 7,000 people (**c**).

In Fig. 14, we compare the proportion of reached population based on static and dynamic view of the population. With the static view, we assume that people are where they sleep (i.e., night-time population) which is a typical assumption in most studies, whereas with the dynamic view of the population we estimate the number of people in the shop’s proximity based on the mobile phone data. Introducing a dynamic population to the analysis matters, as in some areas, the static population underestimates the reached population (Fig. 14a), whereas in other areas it overestimates the reached population (Fig. 14b).

**Fig. 15:**
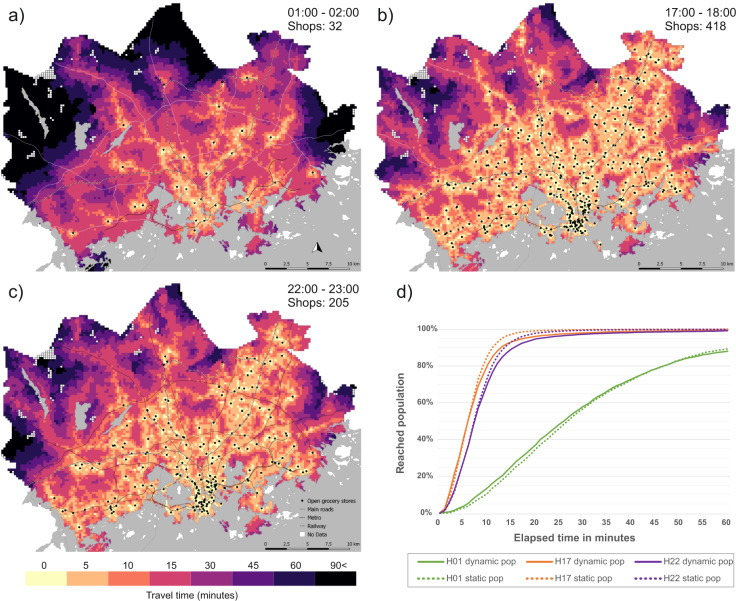
Accessibility to the closest grocery shop (n = 418) using public transport between (**a**) 01:00–02:00, (**b**) 17:00–18:00, (**c**) 22:00–23:00, (**d**) shows the cumulative sum of population reaching the closest grocery shop by public transport.

The mobility dataset presented here also makes it possible to analyse dynamic accessibility of a service network as a whole. At the level of the whole study area, there is little difference in the cumulative number of people that can reach the closest grocery shop between static and dynamic population data. Regardless of the time of day, the difference in the proportion of reached population is less than 10% between the two datasets, although the static population data tends to overestimate the accessibility of the closest shop on time distances above 10 minutes during the daytime (Fig. 15). The network of open grocery shops is dense and widely distributed across the study area both during the day and night with 32 grocery shops open round the clock. Thus, the distance to the nearest grocery shop is constant in the HMA, although the population distribution fluctuates in space.

The accessibility of the closest grocery shop is poorest during the night-time (Fig. 15a,d), when the service level of the public transport network is at its lowest. Thus, the areas with the best accessibility are generally within walking distance of an open shop. Regardless of this, approximately 26% of the population in HMA can access the closet grocery shop within 15 minutes, based on the dynamic population data and 23% based on the static population data. Overall, the accessibility of grocery shops is good during the day. During the daytime, most parts of the study area belong to the 10-minute accessibility zone (Fig. 15b,d). The accessibility gradually starts to decrease after 21:00, when the number of open shops and public transport service level decreases. Between 22:00–23:00, the accessibility of grocery shops is almost as good as during the daytime (Fig. 15c,d) as the proportion of reaching people remains very high (93%), although over 50% of the shops are closed. Based on these examples, it is evident that the difference in grocery shop accessibility between hours is mainly caused by the variation in public transport supply and opening hours of shops, and less due to variation in locations of people.

## Data Availability

The developed codes and tools for generating and validating the population datasets are written in Python and openly available on GitHub: https://github.com/DigitalGeographyLab/mfd-helsinki.
